# Applications of methods of psychological support developed for astronauts for use in medical settings

**DOI:** 10.3389/fphys.2022.926597

**Published:** 2022-09-14

**Authors:** Ivan A. Rozanov, Oleg Ryumin, Olga Karpova, Dmitry Shved, Alexandra Savinkina, Polina Kuznetsova, Nicole Diaz Rey, Ksenia Shishenina, Vadim Gushin

**Affiliations:** ^1^ Russian Federation State Scientific Center, Institute of Biomedical Problems of the Russian Academy of Sciences, Moscow, Russia; ^2^ Moscow Aviation Institute, National Research University, Moscow, Russia; ^3^ M. V. Lomonosov Moscow State University, Moscow, Russia

**Keywords:** psychological support, isolation, monotony, sensory deprivation, social deprivation, COVID-19, countermeasures, virtual reality

## Abstract

Over the past 40 years, psychological support (PS) for cosmonauts and astronauts has remained an important part of the regular biomedical provision of space crews during extended orbital flights. It includes well-developed principles and a set of methods that have proven its effectiveness for the maintenance of behavioral health under extreme conditions of space flight. The main principle of PS in flight is to restore the usual sensory input to compensate for the monotony and lack of external stimuli as a result of a long stay under isolation and confinement. Risk factors for the psychological health and well-being defined for the astronauts, such as sensory and social deprivation, monotony, confinement, and lack of privacy, also remain part and parcel of several civil professions. These include polar wintering, submarines, working on oil platforms, and ocean fishing. Most of these factors also adversely affect the recovery rate of a large contingent of medical institutions, especially bedridden patients with chronic diseases. Finally, due to the negative epidemiological situation associated with the spread of COVID-19, an increasingly wide range of citizens forced to be in self-isolation faces negative manifestations of the deprivation phenomena described previously. Several cases of successful use of PS under isolation, monotony, crowding, and confinement are presented. Thus, we assume that the use of psychological support methods developed for space flights could be extremely relevant in civil medicine and everyday life.

## Introduction

The crews of long-term space expeditions live and perform complex operator activities under conditions of constant exposure to adverse factors of space flight, which pose a real threat to life and health. To counteract the adverse factors of space flight, Soviet specialists in the 70s of the 20th century developed a system of measures for psychological prevention and correction, conventionally named “psychological support”. According to Kozerenko et al. (2001), psychological support (PS) for space crews is defined as a set of psychological methods, tools, and measures used by the mission control services to preserve the psychological health and mental well-being of astronauts under unfavorable conditions of extended space flight. The psychological support system was built in such a way that each group of unfavorable factors of space flight is opposed to a corresponding system of preventive measures (Gushin et al., 2020).

Being in orbit is associated with a prolonged stay in microgravity in a confined small chamber environment. This deprives the astronaut of the usual influx of visual, auditory, and tactile stimuli of the natural terrestrial environment, as well as a complex of vestibular signals associated with the effects of planetary gravity (Kanas, 2015). Along with the decrease in the volume of incoming information in flight, its diversity also decreases. It is caused not only by the monotonous environment created by life support systems but also by the flight protocol with frequent repetition of stereotypical actions for the maintenance of station systems (Kanas et al., 2009). Therefore, the appearance of psychological problems is associated with the processes of asthenization of the central nervous system, progressing decrease of its tone under sensory deprivation and causing inadequate reactions to incoming stimuli (Myasnikov and Stepanov, 2002). Its manifestations were vividly described for astronauts and polar winterers and could lead to increasing fatigue, deterioration of cognitive functions, stereotyping of actions, the appearance of anxiety, boredom, and loss of motivation and morale (Palinkas and Suedfeld, 2008). Psychological support measures for reconstruction of the usual sensory influx include prevention of the occurrence of sensory deprivation caused by the combined effect of sensory deprivation and monotony. They include regular provision of the crew members with the additional sources of external stimuli like fresh news, books, movies, music, and videos from Earth.

Another important group of unfavorable factors of long-term space flight is defined in the works of M.A. Novikov and his colleagues as the imposed nature of communication and the restriction of social contacts (Novikov, 1970). In flight, crew communications are an element of professional activity and are associated with the mandatory informing of the MCC about the life of the crew and receiving instructions and recommendations. An increase in the volume of mandatory professional communication is combined with a decrease in the number and duration of contacts with the cosmonaut’s inner social circle. A person in orbit may lose part of the usual social support from relatives and friends. As a result, a feeling of loneliness and isolation from the usual environment appear, sometimes having an adverse effect on the family life of an astronaut. (Suedfeld, 2018). The conditions of crowding, combined with a high level of stress, can provoke conflict tension in a small group, a struggle for leadership in the crew. Therefore, another area of psychological support is the preservation of the usual circle of communication, maintaining the social ties of the crew in flight. It is maintained by the PS specialists who organize regular video conferences with families, friends, and VIPs for the astronauts (Johnson, 2010). Also, mission controllers are aware that crew members could transfer anger and aggression outside to prevent conflict within the crew (Kanas et al., 2009). They are ready for this draining of negativity and could even provoke it intentionally, to decrease psychological pressure within the crew.

Space and polar expeditions also lead to the fact that crews are forced to stay under conditions of crowding and lack of privacy. It should be noted that crowding and lack of privacy are problems relevant both for staying under lockdown conditions (due to a new coronavirus infection) and for a number of extreme professions (for example, employees of oil rigs in the open sea)—and for the modern urban environment as a whole.

At orbital stations, the problem of lack of privacy is solved by allocating personal spaces (cabins) for astronauts, but such solutions are limited by a shortage of resources and a small habitable volume of orbital stations.

Social isolation and restriction of freedom, as well as the phenomenon of crowding, contradict the socio-psychological nature of man. As a rule, people face similar conditions in exceptional situations. Closed space and limited group interaction can be observed while working on submarines, on Arctic and island expeditions, and in spacecraft and stations, as well as during stay in places of imprisonment and hospitalization. Research conducted back in the 1960s and 70s by I. Altman showed that for people who stay in small residential areas for a long time, the territoriality of behavior is characteristic, and violation of boundaries or rules of behavior in a group provokes conflict situations. Studying the behavior of people under conditions of crowding, I. Altman introduced the concept of privacy, which means “selective control of access to oneself” achieved by modifying the environment, which includes both physical and socio-cultural components. Privacy is ensured through various forms of verbal and nonverbal behavior. Later, in the course of an experiment simulating the work of astronauts under conditions of extreme crowding, the phenomenon of the transition of personal intersubjective territory into the category of interpersonal space was revealed, while the problems of living together in these conditions are solved by personalizing individual and group territories. Further studies conducted, in particular, on densely populated urbanized areas, confirmed the relevance of psychological problems associated with crowding, violation of psychological boundaries, and lack of privacy. The predominance of a negative psycho-emotional background (an increase in the level of aggression) develops as a result of the difficulty in realizing the normal, physiologically conditioned, human need for personal space (the need for territoriality), as well as a result of physical violation of personal boundaries, lack of space, and isolation. The results of the research study show that crowding and high social density can give rise to a feeling of anxiety in a person and worsen mood (Saegert, 1975). At the same time, men experience more negative emotions under conditions of increased density than in situations of reduced spatial density, whereas reduced density has a more depressing effect on women (Freedman et al., 1972). Studies show that prolonged stay of men in space with high crowding increases the level of cortisol in the blood (Heshka and Pylypuk, 1975).

Thus, in isolation, stable asthenization is formed under the influence of sensory deprivation, monotony, the forced nature of communication contacts, increased inactivity, and hypokinesia. It is quite natural that a person’s emotional reaction to isolation and crowding is usually sharply negative.

Usually, several terrestrial models are used as analogs of space flight, like polar expeditions or chamber isolation studies (Palinkas et al., 2000). Vice versa, we describe several simulations and models that are commonly used in space medicine as analogs for the civil medicine cases and practices in the following sections. The objective of this study is to confirm the effects of space PS under conditions that simulate both space flight factors and medical conditions and the practice of civil (terrestrial) medicine.

## Experimental models and research results

### Model 1—dry immersion

Dry immersion (DI) is one of the most widely used ground models of microgravity. DI accurately and rapidly reproduces most of the physiological effects of short-term space flights. Within this model, healthy volunteers are placed, individually or in pairs, in a supine position in a covered bath that was filled with water at a temperature of 33 ± 0.5°C. The daily routine is specified in accordance with the schedule of studies and countermeasure procedures (if the experiment included them), including 8 h of sleep, 3–4 meals, a medical supervision program, and experimental studies. The model simulates such factors of space flight as mechanical and axial unloading as well as physical inactivity (Tomilovskaya et al.., 2019). We regard dry immersion as a perfect simulation of staying of bedridden patients in the hospital (i.e., after surgery). In both cases, subjects are limited in motor activities, sensory and socially deprived, and have problems with organization of their free time.

The negative impact of hypokinesia on mental health both under the conditions of model experiments including DI and extended stay in the hospital and under the conditions of space flight arises as a result of a discrepancy between the afferent (sensitive) link of the central nervous system under load and another link (effector, i.e., motor), limited by the load of the general functional chain of motor acts. Negative, subjectively “difficult” mental states perceived by recipients may be expressed in neurotic manifestations. In particular, during the period of acute adaptation to the conditions of the experiment, irritability, complaints, increased conflict tension with the experiment staff, increased attention to one’s own proprioceptive sensations (including pain), sleep disturbances at the stage of adaptation to dry immersion, boredom, and problems with organizing free time can be observed (Rozanov et al., 2022).

In October–December 2020, a 3-day dry immersion with the participation of six women from 24 to 39 years old (the average age is 30 years) was carried out on the basis of the IMBP RAS. The study was approved by the Commission on Biomedical Ethics of the IMBP RAS (Protocol No. 544 dated 06/16/2020). The recipients signed informed consent. It should be noted that the duration of immersion exposure (3 days) allowed us to consider this entire time interval as a period of acute adaptation to stressful experimental conditions. In the 3-day dry immersion aimed at studying the physiological and psychophysiological state of the women’s body under the conditions of modeling the factors of space flight, the method of psychological support based on VR technologies was tested.

The objective of our study was to test the PS complex based on VR technologies and to study the psychophysiological effects that occurred in the recipients when they interacted with this complex. In order to meet the safety criterion, preliminary training of the subjects was conducted. According to the data of medical (neurological) examinations after VR sessions, no violations were detected. The psychological safety of the content was ensured through the preliminary censorship of audiovisual materials. The personification of the presented content was achieved through a preliminary questionnaire about preferences and the subsequent formation of a “repertoire” based on their analysis. The principle of matching the correspondence of the objective function was applied. Autonomous, i.e., not requiring connection to an external computer, VR helmets with properties such as lightness of construction, mobility, and ease of use were used. In addition, this “repertoire” was compiled taking into account the nature of the impact of the experimental conditions: immobilization, staying in an unsupported position, prolonged (almost constant) staying in a supine position, redistribution of body fluids, exceptionally small sensory influx through a number of sensory systems, and partial social deprivation (narrowing of the circle of social contacts and the predominance of forced and professional communication). Thus, to prevent active locomotion while perceiving visual images, presented pictures should simulate flying or gliding in an environment with low resistance, like atmosphere or water. As a VR environment, we used three-dimensional video, i.e., the content was non-interactive, not requiring active movements and other motor activity to interact with it—which is also necessary from the point of view of maintaining the purity of the experiment. Based on this, the content of the videos included flights above the planet’s surface (both characteristic of the place of residence of the subjects and exotic) and swimming in the sea among marine animals (Rozanov et al., 2021; Rozanov et al., 2022).

We used non-invasive and objective research methods to assess the psychophysiological effects of PS. The participants recorded semi-structured self-reports about their health, mood, and working capacity. They were analyzed using Noldus FaceReader software and speech content analysis to get the data about their emotional status and cognitive functions (Kьntzler et al., 2021). With the help of this complex, the severity of the emotionality of the subjects was assessed in comparison with a calm facial expression. FaceReader software is able to evaluate the following indicators based on the facial expressions of testers: neutral (lack of emotions), valence and arousal, and emotions such as happy, sad, angry, surprised, scared, and disgusted. This hardware and software complex, as well as the corresponding computerized method of analysis, is an objective, validated method, widely implemented in the practice of psychological research. Actigraphy was applied to analyze subjects’ locomotion and sleep duration by sleep analysis using the Cole–Kripke algorithm (Cole et al.., 1992), calculations based on which were carried out using ActiLife software. Also, specially developed questionnaires were used to obtain the data about subjects’ perception of the level of PS provided by the on-duty team (doctor, technician, and laboratory assistant) and the VR sessions. The questionnaire “Immersiveness” was developed in order to evaluate the subjective “presence effect” that occurs in testers during a virtual reality session (synonyms—immersion effect, immersiveness).

For statistical analysis of this model study, methods such as the calculation of the Wilcoxon and Spearman criteria were used, while the computer analysis was carried out in the SPSS program.

Results. In the self-reports during immersion, subjects, as expected, often complained about increasing fatigue caused by a “tightly” compiled protocol of studies, boredom, and sleep disorders. These types of complaints were mentioned in 23.3% of reports. Also, discomfort associated with difficulties in performing urination and defecation was mentioned. To conclude, staying in dry immersion in general negatively affected their psycho-emotional state.

According to the data of medical observation of the subjects after the VR sessions, four of them had a slightly pronounced pendulum-like fine-grained binocular mixed nystagmus of the first degree that can be regarded as physiological, not pathological, nystagmus. Two recipients did not report nystagmus at all. Nystagmus took place for 0.5–1.5 min after the end of the VR session. During the use of VR technical means and the selected visual content, no cases of nausea, dizziness, motion sickness, or coordination disorders were detected in the subjects who were under the conditions of DI.

To assess the impact of VR sessions on the cognitive and emotional spheres, detection of speech errors and filler words (or hesitation marks, which are so-called in linguistics as discursive and are markers of stress) in the transcripts of self-reports was carried out. It was found that the proportion of parasite words and speech errors in self-reports after the VR session decreased significantly (T = -2,201; *p* < 0.05). So, according to self-reports, a positive effect of VR sessions on the cognitive functions of the subjects was also found (Rozanov et al., 2022).

To estimate changes in the psycho-emotional status during dry immersion, a comparison of the average values of basic emotions was made using FaceReader. Facial expressions were recorded during DPC (daily planning conference, afternoon planning conference in the form of a structured self-report) of the recipients as well as before and after the VR session. A statistically significant tendency to increase the proportion of the neutral component in facial expressions was found (t = 3,155; *p* = 0,008). After the VR session, the proportion of neutral components in facial expression increased even more and was also statistically significant (T = 2,605; *p* < 0.05). At the same time, the indicator of the level of arousal statistically significantly decreased after the sessions (T = -0.973; *p* < 0.05). In other words, they were looking calmer and less tensed throughout the stressful conditions that can be an indicator of gradual adaptation to the experimental conditions. From that point, the impact of VR can be regarded as relaxing.

The effect of utilization of non-interactive VR content was also manifested in a decrease in the general volume of motor activity during the PS session. That was confirmed by the fact that the maximum of the motion vector during VR sessions was on average 2.4 times lower than the maximum per day. The maximum of the motion vector after the VR session was less in 13 out of 15 cases, 1.3 times on average (T = -2.48; *p* < 0.05). We suppose that a pronounced decrease in motor activity (and corresponding more pronounced tonic relaxation) during dry immersion also could be related to the facial expression of the subjects. For example, we detected an increase in the level of happiness emotion found by FaceReader before and after VR, which negatively correlated with the ratio of the daily maximum motor activity (VM) (с = -0.741; *p* < 0.01). There was also a stable positive correlation between the degree of immersion in visual virtual images and the difference in the severity of the arousal emotional index before and after the VR session (с = 0.598; *p* < 0.05). This correlation suggests that more psychologically immersive images of VR, in all likelihood, caused a more pronounced decrease in the “arousal” index in the subjects.

The obtained results allow us to draw a preliminary conclusion about the general relaxing and calming effect of PS via VR that reduces mental tension caused by adverse psychological factors of dry immersion.

### Model 2—isolation experiment

The COVID-19 pandemic has become one of the most severe crises for public health and society in recent times. With many adverse consequences, such epidemics are always associated with adverse consequences for mental health (Palinkas et al.., 2021). The critical reduction of sensory information may be also explained by the reduction of social contacts, which decreases the chance of additional verification of social reality (Alle and Bernsten, 2021). It can make it difficult to distinguish between reality and imagination, which leads to distortions of perception, derealization, and hallucinations. There are reasons to believe that social isolation associated with confinement and monotony under quarantine can have important psychological consequences in terms of increased psychotic symptoms and cognitive problems (Fiorillo and Gorwood., 2020).

Such problems were noticed in 2020 in Italy during the second month of national isolation (D’Agostino et al.., 2020). Patients without a psychiatric history could have hallucinations and a somatic illusion that they were infected with the SARS-CoV-2. That corresponds with the old isolation study made in the middle of the 20th century by the McDonnell Douglas Corporation (Gushin, 2020). A depressing psychological state is also caused by uncertainty about the future, which is associated with a lack of data about the time of complete recovery from the disease on a global scale, as well as with a large number of uncontrolled news. That is why researchers express concern about the negative impact of self-isolation on both individuals who are forced to face prolonged isolation for epidemiological or medical reasons and patients with mental illness, whose clinical outcome may occur with the risk of exacerbation of symptoms and possible relapse (Chaturvedi, 2020).

A short-term isolation (chamber) experiment can be regarded as a short quarantine of the family group or small professional group that was typical for the COVID pandemic.

The ESKIS experiment with 14-day isolation and crowding of the mixed gender crew was carried out in the chamber of 50 cubic meters at the ground-based experimental complex (NEK) SSC RF–IMBP RAS. Six practically healthy volunteers, four men and two women (age 23–45 years), participated in the experiment. Five of them had no previous experience in isolation experiments (Gushin et al., 2020; Nosenkova, 2021). The daily routine was specified in accordance with the schedule of studies, including the execution of operator’s performance tasks, three meals, 8 h of sleep, a medical supervision program, and experimental studies. The main psychological factors affecting the subjects during the experiment are typical for chamber confinement studies (as well as isolation caused by epidemiological reasons): sensory deprivation, monotony, crowding, lack of privacy, the limitation of social contacts, and the forced nature of communication (Feichtenger et al., 2012). It is important to note that subjects executed performance tasks, which corresponds with a remote regime of working that was established during the pandemic.

The PS complex included PS sessions based on VR technologies and sessions of “classical” psychological support, similar to those used in orbital flights. These included providing crew members with personalized multimedia content to view on personal tablets. This content was served by videos of a documentary nature and relaxation orientation with views of nature or space (for the creation of a “flight image” for the crew members) and also art therapy sessions, which included drawing on suggested topics and coloring books for adults. Sessions of each type of PS were conducted four times during isolation for each crew member in accordance with the cyclogram of the experiment.

For VR-based PS sessions, a special hardware and software complex was provided based on an autonomous portable VR helmet, and special software for this helmet was developed (and tested for the first time under extreme conditions in this experiment). This software was provided by IBMP specialists in cooperation with AI Health LCC. It allows users to transfer to a virtual analog of the personal space: with the help of VR tools, an interactive image of a personal recreation room is created. In this virtual room, the user can make changes to the interior, change the weather and time of day outside the window of the room, and control the fireplace according to personal preferences. A set of personal multimedia content is available from this room for listening and viewing.

We supposed that subjects, who for the first time faced a complex of factors of isolation in a hermetic object, lack of personal space, and crowding, may experience anxiety—a state associated with a sense of uncertainty (Gushin, 2020). According to space practice, PS under conditions of extreme confinement, crowding, and isolation can be obtained *via* contact with mission controllers (in this study, duty medical teams) or within the crew. Social support inside the cohesive crew is based, according to Weiss, on the “availability of people you can rely on” (Sarason et al., 1990). Another source of PS included the provision of personalized multimedia content to compensate sensory deprivation and monotony and organize leisure time. A VR complex was also used, identical to the one used in dry immersion.

We expected that subjects with better developed communicative skills and extroverted personalities would obtain PS mostly *via* contacts with the crew and supporting group. On the other hand, subjects with dominating introversion could have problems with getting social support to withstand sensory deprivation, monotony, and crowding. For them, PS *via* computerized content, including the virtual reality environment, which is clearly dosed, contains preliminarily defined relaxing data and provides an additional virtual space, should be the most preferred type of psychological support.

The research methods were similar to those in 3-day dry immersion. Also, Keirsey Temperament Sorter (KTS) was utilized to estimate the influence of personality traits on the preferences of PS type. The questionnaire “psychological support”, which was also used in the 3-day DI, was supplemented for this experiment with new graphs that allow us to assess the degree of support from communication with the crew and a sense of association with it. This change was dictated by the fact that in the isolation experiments, contrary to dry immersion, participants have contacts not only with the representatives of the duty teams but also within the crew (Sholcova et al., 2021). To study the social interactions in the crew and the cohesion of the crew, we applied a method for diagnosing the value-oriented unity of the group.

For statistical analysis in these model studies, we used the same methods as in the dry immersion model experiment.

Results. Undercrowding conditions, the indicator “extraversion” obtained using KTS had a positive stable correlation with the indicator of support from communication (i.e., social support) with the duty crew (*p* = 0.896, *p* < 0.05), and the indicator “introversion” had a negative relationship with the same parameter (*p* = -0.896, *p* <0.05)—according to the questionnaire “psychological support.” Thus, it can be assumed that the extroverted crew members subjectively experienced a higher level of support from communication (which corresponds to such an event of “classical” psychological support as the organization of private communication channels) than from instrumental methods of psychological support.

By analyzing the data of the Keirsey test, it was found that persons with a tendency to introversion, who did not feel unity with the crew and the need to communicate with the duty crew, were more susceptible to psychological support using virtual reality technology. The indicator “extraversion” obtained using the Keirsey questionnaire had a positive stable correlation with the indicator of support from communication with the duty crew (r = 0.896, *p* < 0.05), and the indicator “introversion” had a negative relationship with the same parameter (r = -0.896, *p* < 0.05). It should also be noted that the average values of communication positively correlated with both the degree of association of the subjects with the crew (perception of themselves as part of the group) and the level of support (r = 0.888, *p* < 0.05 and r = 0.858, *p* < 0.05, respectively). In addition, it was shown that the indicator of the need for generalization and interpretation of information correlated negatively with support from BP (r = - 0.858, *p* < 0.05). In addition to the indicator of introversion, the indicator of the need for specific information (sensation index according to Keirsey) positively correlated with the need for PS based on VR (r = 0.888, *p* < 0.05). Thus, it can be assumed that introverted crew members had a higher expressed need for such an instrumental form of psychological support as VR. The facial expression analysis also showed a reduction of the emotion of anger after the session in all subjects (T = -1,78, *p* = 0,89)).

A positive two-way correlation was also established between photo support and sympathy for the duty crew (r = 0.852, *p* < 0.05) and between photo support and reading support (r = 0.865, *p* < 0.05). We assume that under the conditions of narrowing the circle of social contacts inherent in isolation, such types of content as photos and books performed a socially substitutive role in the surveyed.

Actigraphy data indicate a deterioration in the quality of sleep in isolation. In all the subjects, except one, the duration of sleep steadily decreased during the experiment. All subjects, except one, had a tendency to decrease the time spent in bed during the night hours allotted for sleep, while all subjects, except two, had an increase in the number of night awakenings. According to actigraphy data, the use of psychological support using VR technologies could cause a decrease in the number of nocturnal awakenings (by 1.3–1.4 times compared to the average value for the experiment) and an increase in sleep duration (by 10–13.3% relative to the average value for the experiment).

### Limitations of the study

As we have already pointed out, these models are quite correlated with a wide range of medical problems faced by physicians and clinical psychologists on Earth. But significant limitations of this study are associated with the small sample size and short duration of model experiments. However, 1) this situation is typical for model experiments that are difficult to organize, and 2) our research is one of the first objective studies of the psychophysiological effects of psychological support. Previously, the effectiveness of PS was confirmed solely on the basis of subjective self-reports of astronauts and the opinions of psychoneurologists supervising them during the flight. Nevertheless, work in this direction continues both in a number of new model experiments and in control groups. Also, the available objective scientific data presented in this article confirm the effectiveness of promising types of PS relevant for implementation in healthcare. The description of the planned scope of the introduction of PS methods is based on a systematic analysis of a wide range of scientific literature.

## Expected scope of application of psychological support methods

It seems promising and expedient to apply psychological support methods for orbital space flights in a number of extreme professions, whose representatives are characterized by a long exposure (exposure) of risk factors similar to those during space flight. It should be noted here that these measures can be applied as part of a whole set of PS for representatives of extreme professions who have been under extreme conditions for a long time, in isolation, combined with a number of other factors, for example, crowding and monotony, or separately, during off-duty hours, to correct the negative impact of stress factors on the body and mind, for psychoprophylaxis and psychohygiena.

1) Conditions of social isolation, sensory deprivation, and monotony are a typical set of unfavorable psychophysiological factors accompanying the long-term stay of patients in hospitals. Having been immobilized for a long time, deprived of contact with nature and mundane environment stimuli, and with a narrowed circle of social contacts, such patients are increasingly plunged into depression and do not show active volitional efforts for a speedy correction. Under these conditions, the familiar visual and auditory environment reconstructed with the help of computerized content, including virtual reality technologies (pictures of nature, native homes, and art objects–museums and art galleries) can create an additional information influx compensating for sensory deprivation. Thus, the detrained nervous system can restore its activity. This will have a positive effect not only on the mood but also on the well-being of bedridden patients and stimulate the desire to return to a full and normal life, i.e., to recover.
2) Social security and patronage of the elderly and sedentary disabled are currently virtually devoid of any means of psychological support. A system of psychological support based on elaborate space psychology tools and methods can be used with the participation of social security personnel. It can expand the range of services provided to the elderly and disabled and improve their life quality.
3) Conditions of prolonged quarantine caused by the epidemiological situation, with prolonged stay in monotonous confinement and isolation conditions with social and sensory deprivation, negatively affect the tone of the central nervous system (Li et al., 2022). Similarly to the previous set of conditions, providing quarantined people with means of PS will favorably affect the functioning of the central nervous system and compensate unfavorable effects of extended sensory and social deprivation.
4) Autonomous performance under extreme living conditions (polar wintering and remote drilling, etc.) is in many ways similar to the conditions of long-term space flight in terms of a set of factors adversely affecting humans. Workers are affected by the same sensory and social deprivation and monotony (Suedfeld, 2012). As a result, in particular, there is an increase in depression, sleep disorders, and conflict tension in winter quarters, which adversely affects not only the state of health but also labor productivity.
5) Personnel of fishing vessels engaged in fish processing, which is characterized by sensory “hunger,” being in crowded conditions and extremely pronounced monotony of activity (monotony).
6) Divers undergoing prolonged decompression in a pressure chamber, which is characterized by sensory hunger, monotony, the inability to organize active forms of recreation, difficulties with structuring leisure, and a decrease in intellectual (cognitive) abilities against the background of natural physiological processes inherent in decompression.

It should be noted here that the described measures can be applied partially or as a whole complex of PS to correct the negative effects of stress factors on the body and psyche, as an effective set of countermeasures.

The criteria for the application (applicability) of PS methods should include:

1) health safety;
2) psychological safety—the content and the stimuli provided should not contain audiovisual stimuli that cause fear, anxiety, and other negative emotions and mental states in recipients;
3) personification—the content and the stimuli provided must be individually formed taking into account the personal cultural needs of the recipient, as well as his current psycho-emotional state;
4) compliance with the objective function—prevention of the adverse effects of psychological factors of the altered environment (i.e., space flight, ground-based model experiment, autonomous activity in isolation, and quarantine), in which the use of the PS complex is planned.

In the case of developing a set of PS measures for representatives of a certain type of extreme professions, psychological risk factors inherent in a particular profession should be taken into account. In accordance with the principle of compliance with the target function, the developed set of PS measures should include specific methods aimed against specific risk factors, taking into account their specific weight.

In addition, it is necessary to take into account the expected exposure time (exposure time) of a factor and the expected duration of stay under altered, extreme living conditions. This may be, for example, due to the fact that the amount of multimedia content for “classical” psychological support should be determined in such a way that it is enough for persons undergoing psychological support for the duration of the entire isolation without the development of such effect—when users are bored by the PS method or content provided via PS session. For example, VR in dry immersion, according to the data presented previously, served as an effective countermeasure against sensory hunger. However, it is obvious that the provided set of relaxation videos will not be enough for long-term isolation, since the video repertoire was prepared with the expectation of an experiment duration of 3 days. At the same time, the available literature data on the group dynamics of psychophysiological indicators of persons in isolation should also be followed. These data can serve as a kind of predictive model. For example, a period of 3–14 days can be characterized as a period of acute adaptation to extreme, unusual, altered living conditions. Also, thanks to the experience of isolation experiments, the so-called phenomenon of the third quarter has become well known—increasing asthenization, conflicts within the crew, and a decrease in motivation to perform a flight task, combined with a loss of interest in interacting with external subscribers, which falls precisely on the third quarter of isolation (accordingly, having this information when planning a set of PS measures, for example, for a remote expedition, it is possible to work out a proactive set of measures timed precisely to the third of the expedition period).

It is necessary to take into account not only the totality and peculiarity of risk factors and the gender and cultural characteristics of the representatives of the small group for which the PS complex is being developed but also technical features, primarily related to the habitable volume of the small group’s place of residence and the bandwidth of communication channels. It is obvious that an acute shortage of habitable volume will make it impossible to apply recommendations on active forms of leisure and sports for psychological recreation (this was modeled in the ESKIS experiment, and active games in a virtual reality environment served as a countermeasure to such an acute shortage of motor activity). If there are significant communication delays, irregular communication sessions, and low bandwidth of information exchange channels, it is necessary to take into account the complex of psychological support methods being developed for interplanetary flights and adopt from it a number of methods applicable under conditions of increased autonomy.

## Conclusion

During two simulated studies, a new hardware and software complex developed for providing PS sessions underwent medical testing within the framework of the activities of the Pavlov Center for Integrative Physiology for Medicine, High-tech Healthcare, and Stress Tolerance Technologies. Observed psychophysiological changes in the psychophysiological status of subjects under stressful conditions allow us to state that PS measures, elaborated for the needs of space medicine, effectively provide psychological support for the people experiencing prolonged stress, caused by confinement, sensory deprivation, monotony, and social isolation. Obtained results give us the opportunity to recommend applying psychological support methods, elaborated for orbital space flights, for the needs of terrestrial medicine.

## Data Availability

The original contributions presented in the study are included in the article/Supplementary Material; further inquiries can be directed to the corresponding author.

## References

[B1] Allé M. C. Berntsen D. (2021). Self-isolation, psychotic symptoms and cognitive problems dur-ing the COVID-19 worldwide outbreak. Psychiatry Res. 302, 114015. 10.1016/j.psychres.2021.114015 34062477 PMC8131183

[B3] Chaturvedi S. K. (2020). Covid-19, coronavirus and mental health rehabilitation at times of crisis. J. Psychosoc. Rehabil. Ment. Health 7 (1), 1–2. 10.1007/s40737-020-00162-z 32292688 PMC7114947

[B4] Cole R. J. Kripke D. F. Gruen W. Mullaney D. J. Gillin J. C. (1992). Automatic sleep/wake identification from wrist activity. Sleep 15 (5), 461–469. 10.1093/sleep/15.5.461 1455130

[B5] D'Agostino A. D'Angelo S. Giordano B. Cigognini A. C. Chirico M. L. Redaelli C. (2020). Brief psychotic disorder during the national lockdown in Italy: An emerging clinical phenomenon of the coronavirus pandemic. Schizophr. Bull. 47, 15. 10.1093/schbul/sbaa112 PMC7454891 32761196

[B6] Feichtinger E. Charles R. Urbina D. Sundblad P. Fuglesang C. Zell M. (2012). “MARS-500 – a testbed for psychological crew support during future human exploration missions,” in 2012 IEEE Aerospace conference, USA: Big Sky Montana, March 3–10, 2012.1–17.

[B7] Fiorillo A. Gorwood P. (2020). The consequences of the COVID-19 pandemic on mental health and implications for clinical practice. Eur. Psychiatry 63, e32. 10.1192/j.eurpsy.2020.35 32234102 PMC7156565

[B18] Freedman J. L. Lewy A. S. Buchanan R. W. Price J. (1972). Crowding and human agressivness. J. Exp. Soc. Psychol. 8, 528–548. 10.1016/0022-1031(72)90078-9

[B9] Gushin V. (2020). “Chapter “isolation chamber studies”, encyclopedia of bioastronautics,” in Lawrence R. Young, jeffrey P. Sutton (Berlin, Germany: Springer).

[B10] Gushin V. Shved D. Yusupova A. Supolkina N. Savinkina A. Lebedeva S. (2020). “Features of communication of a crew of mixed national and gender composition with the Control Center under communication delay in SIRIUS-18/19,” in Proceedings of the 71st International Astronautical Congress (IAC), October 12–14, 2020. Paris, France. The CyberSpace EditionIAC-20-A1.1.3.x56843.

[B25] Heshka S. Pylypuk A. (1975). “Human crowding and adrenocortical activity,”. Paper presented at the Canadian Psychological Association. Montreal

[B12] Johnson P. J. (2010). The roles of NASA, U.S. astronauts and their families in long-duration missions. Acta Astronaut. 67, 561–571. 10.1016/j.actaastro.2010.05.001

[B13] Kanas N. (2015). Humans in space – the psychological hurdles. New York: Springer.

[B14] Kanas N. Sandal G. Boyd J. E. Gushin V. I. Manzey D. North R. (2009). Psychology and culture during long-duration space missions. Acta Astronaut. 64, 659–677. 10.1016/j.actaastro.2008.12.005

[B15] Kozerenko O. P. Sled A. D. Mirzadzhanov Y. A. (2001). Psychological support for crews In: Orbital station «Mir. Space Biol. Med. 1, 365–377.

[B16] Küntzler T. Höfling T. A. Alpers G. W. (2021). Automatic facial expression recognition in standardized and non-standardized emotional expressions. Front. Psychol. 2021, 627561. PMID: 34025503; PMCID: PMC8131548. 10.3389/fpsyg.2021.627561 PMC8131548 34025503

[B17] Li N. Fan L. Wang Y. Wang J. Huang Y. (2022). Risk factors of psychological distress during the COVID-19 pandemic: The roles of coping style and emotional regulation. J. Affect. Disord. 299, 326–334. 10.1016/j.jad.2021.12.026 34920036 PMC8683097

[B19] Myasnikov V. I. Stepanova S. I. (2002). Risk factors for developing mental asthenia in astronauts on a long flight. Vestn. TGPU 3, 9–11.

[B20] Nosenkova S. (2021). The module mates. Rus. Kosm. 27, 14–19.

[B21] Novikov M. A. (1970). Communication structure and effectiveness of group activity of operators. Vopr. Psikhol. 4, 130–135.

[B22] Palinkas L. A. Gunderson E. K. E. Johnson J. C. Holland A. W. (2000). Behavior and performance on long-duration spaceflights: Evidence from analogue environments. Aviat. Space Environ. Med. 71(9), A29–A36. 10993306

[B23] Palinkas L. A. Springgate B. Hancock J. Sugarman O. K. Pesson C. L. Stallard C. N. (2021) Impact of the COVID-19 pandemic on resilience to climate change in underserved communities. Sustain. Clim. change 14. 0022. 10.1089/scc.2021.0022

[B24] Palinkas L. A. Suedfeld P. (2008). Psychological effects of polar expeditions. Lancet 371, 153–163. 10.1016/S0140-6736(07)61056-3 17655924

[B26] Rozanov I. A. Kuznetsova P. G. Savinkina A. O. Shved D. M. Ryumin O. O. Tomilovskaya E. S. (2022). Psychological support using virtual reality in an experiment with three-day dry immersion. Aerokosmicheskaya i Ekol. Meditsina Russ. 56 (1), 55–61. 10.21687/0233-528x-2022-56-1-55-61

[B27] Rozanov I. Gushin V. Ruymin O. Karpova O. Shed D. (2021). Psychological support based on virtual reality in simulation experiments, isolation and space flights. Aerosp. Enviromental Med. 55, 114.

[B11] Saegert S. (1982). “Environment and children’s mental health: Residential density and low income children,”. Handbook of psychology and health. Editors Baum A. Singer J. E. (Hillsdale, NJ: Erlbaum), 247–271.

[B28] I. G Sarason B. R. Sarason G. R Pierce (Editors) (1990). Social support: An Interactional view (NY: Wiley), 528.

[B29] Sholcova I. Vinokhodova A. Gushin V. Kuznetsova P. (2021). Anticipated and perceived personal growth and values in two spaceflight simulation studies. Acta Astronaut. 179, 561–568. 10.1016/j.actaastro.2020.11.029

[B30] Suedfeld P. Gushin V. I. (2018). Being a father during the space career: Retired cosmonauts' involvement. Acta Astronaut. 149, 106–110. 10.1016/j.actaastro.2018.05.028

[B31] Suedfeld P. (2012). “Extreme and unusual environments: Challenges and response,” in The oxford handbook of environmental psychology. Editor Clayton S. D. (USA: Oxford University Press), 348–371.

[B32] Tomilovskaya E. Shigueva T. Sayenko D. Rukavishnikov I. Kozlovskaya I. (2019). Dry immersion as a ground-based model of microgravity physiological effects. Front. Physiol. 10, 284. 10.3389/fphys.2019.00284 30971938 PMC6446883

